# Impact of 2003 State Regulation on Raw Oyster–associated *Vibrio vulnificus* Illnesses and Deaths, California, USA

**DOI:** 10.3201/eid1908.121861

**Published:** 2013-08

**Authors:** Duc J. Vugia, Farzaneh Tabnak, Anna E. Newton, Michael Hernandez, Patricia M. Griffin

**Affiliations:** California Department of Public Health, Richmond and Sacramento, California, USA (D.J. Vugia, F. Tabnak, M. Hernandez); and Centers for Disease Control and Prevention, Atlanta, Georgia, USA (A.E. Newton, P.M. Griffin)

**Keywords:** *Vibrio vulnificus*, oysters, regulation, California, bacteria, vibrio, vibrio spp., raw oysters, shellfish, seafood, foodborne infections, vibriosis, Gulf of Mexico

## Abstract

US vibriosis rates have increased since 1996, and many *Vibrio vulnificus* infections are fatal. In April 2003, California implemented a regulation restricting the sale of raw oysters harvested from the Gulf of Mexico during April 1–October 31, unless they were processed to reduce *V. vulnificus* to nondetectable levels. We analyzed California cases of *V. vulnificus* infection before and after the regulation’s implementation and compared case data with data from other states. The annual number of reported *V. vulnificus* infections and deaths in California with patient’s sole exposure to raw oysters dropped from 0 to 6 cases and 0 to 5 deaths per year during 1991–2002, before implementation, to 0 during 2003–2010, after implementation (p = 0.0005 for both). In other states, median annual numbers of similar cases and deaths increased slightly after 2002. The data strongly suggest that the 2003 regulation led to a significant reduction in reported raw oyster–associated *V. vulnificus* illnesses and deaths.

## Abstract 2

TOC summary: After regulation was implemented, the number of cases and deaths dropped significantly; a similar national regulation would likely decrease US infections.

A recent review of surveillance data indicated that rates of *Vibrio* spp. infections in the United States increased from 1996 to 2010, and, of the 3 most commonly reported species, *V. vulnificus* caused the most hospitalizations and deaths (*1*). *V. vulnificus* is a gram-negative, halophilic bacterium that occurs naturally in marine and estuarine waters. Human infection usually results from exposure to the organism by consumption of raw or undercooked shellfish, usually oysters, or by a wound coming into contact with seawater. Illness typically is manifest as primary septicemia (following ingestion) or as wound infection with or without septicemia (following wound exposure) (*2**–**5*). Persons at risk for severe *V. vulnificus* disease are those with preexisting liver disease, alcoholism, diabetes, hemochromatosis, or an immunocompromising condition. Patients with primary septicemia often are in shock when they come to medical attention, and the fatality rate has been reported to be >50% (*3**,**4*). Most patients with primary septicemia report recent consumption of raw oysters, usually from the Gulf of Mexico (*2**–**4*).

Most oysters harvested in the United States are from the Gulf Coast region (*6*). Surveys regarding raw oysters in the US market have repeatedly found that Gulf Coast oysters have higher frequency and levels of *V. vulnificus* bacteria than oysters from the North Atlantic or Pacific Coasts, especially during the summer months (*7**,**8*). However, raw oysters can be treated with a postharvest processing method to reduce *V. vulnificus* to “nondetectable” levels, which is defined nationally as a most probable number of <30 organisms/gm oyster meat (*9**,**10*). Three postharvest processing methods are commercially available: 1) individual quick freezing, by which half-shell oysters were rapidly frozen, 2) mild heat–cool pasteurization, by which oysters are heated in warm water and then dipped them in cold water to stop the process, and 3) high hydrostatic pressure processing, in which oysters are subjected to pressure <45,000 pounds per square inch.

In 1991, California adopted a regulation to decrease oyster-associated *V. vulnificus* infections and deaths. Restaurants and other food establishments that sold or served raw Gulf Coast oysters were required to provide the following written warning to prospective customers about the potential harmful effects of consuming raw oysters: “Eating raw oysters may cause severe illness and even death in persons who have liver disease (for example, alcoholic cirrhosis), cancer, or other chronic illnesses that weaken the immune system.” In 1996, the Los Angeles County Department of Health Services reported that, despite this regulation, *V. vulnificus* cases and deaths due to eating raw oysters were ongoing, especially among the Spanish-speaking Hispanic population. A survey of 103 restaurants serving raw Gulf Coast oysters showed that >50% either had no warning sign or a poorly visible sign (*11*). In 1997, California updated the raw oyster regulation to require provision of the written warning both in English (“Warning”) and in Spanish (“Aviso Importante”), with specific wording and formatting requirements for a prominently posted sign, a boxed statement prominently placed on each menu, or a tent card for each dining table (*12*). 

Despite implementation of these updated regulations, oyster-associated *V. vulnificus* infections and deaths continued. This situation led the state of California to enact an emergency regulation on April 14, 2003, restricting the sale, in California, of raw oysters harvested from the Gulf of Mexico from April 1 through October 31, unless the oysters were treated with a scientifically validated process to reduce *V. vulnificus* to nondetectable levels (defined for California as <3 most probable number of organisms/gm/oyster meat) (*12*). California is the only state with this restriction on the sale of raw summer Gulf Coast oysters. 

To assess the public health effects of the 2003 California emergency regulation, we analyzed records for California cases of raw oyster-associated foodborne *V. vulnificus* infection before (1991–2002) and after (2003–2010) implementation of the regulation. We then compared the data with data for cases reported from other states.

## Methods

*Vibrio* infection surveillance in the United States was initiated in 1988 by the Gulf Coast states of Alabama, Florida, Louisiana, and Texas, the US Centers for Disease Control (now US Centers for Disease Control and Prevention [CDC]), and the US Food and Drug Administration. By the early 2000s, most states were reporting cases of *Vibrio* infection to CDC’s Cholera and Other *Vibrio* Illness Surveillance (COVIS) system, and in 2007, vibriosis became nationally notifiable. For each case, information collected on the COVIS form includes demographics, clinical symptoms, underlying illness, history of seafood consumption, exposure to seawater, and *Vibrio* species. In California, *Vibrio* infections have been reportable since 1988 (and the same COVIS form has been used). When shellfish exposure is reported, the local environmental health specialists and the Food and Drug Branch of the California Department of Public Health attempt to trace back the shellfish to its harvest site.

Cases reported to COVIS are classified into foodborne, nonfoodborne, or unknown transmission routes on the bases of the reported exposure (seafood consumption, marine/estuarine water contact, unknown) and specimen site (gastrointestinal, blood, or other normally sterile site; skin or soft tissue, other nonsterile site; unknown). We defined a case as foodborne if the patient reported seafood consumption as the only exposure. We also considered cases foodborne if both of these conditions are met: 1) the exposure is unknown or the patient reported seafood consumption and exposure to marine/estuarine water, and 2) *Vibrio* isolates were obtained only from a gastrointestinal site or from multiple sites, including a gastrointestinal site but not a skin or soft tissue site.

We examined reports from 1991 to 2010 of California cases of oyster-associated *V. vulnificus* infection for patient’s death, age, sex, race/ethnicity, history of liver disease, or alcoholism or other underlying conditions, and for oyster preparation and harvest site. We initially examined the large group of cases in patients who consumed any oysters, raw or cooked, with or without other seafood, with mode of transmission classified either as foodborne or as unknown (e.g., because the patient had both food and water exposure and only a blood isolate). We then narrowed the analysis to only foodborne cases among patients who reported consuming only raw oysters.

For comparison, we examined reports from 1991 to 2010 of cases of foodborne *V. vulnificus* infection from the rest of the United States for resulting death and oyster harvest site, focusing on cases among patients who reported consuming only raw oysters. Data were analyzed by using SAS software, version 9.1 (SAS Institute, Inc., Cary, NC, USA). We used the Wilcoxon-Mann-Whitney 2-sample test to compare the distribution of the annual number of cases before (1991–2002) and after (2003–2010) implementation of the 2003 emergency regulation.

## Results

During 1991–2010, California reported 88 patients with *V. vulnificus* infection. Among them, 61 (69%) had a history of eating any oysters, raw or cooked, with or without other seafood, in the 7 days before illness began and had a mode of transmission classified as foodborne or as unknown. Thirty-nine (64%) of these patients died. The median annual number of cases dropped from 5.5 (range 1–9; total 57 cases) during 1991–2002, before implementation, to 0 (range, 0–2; total 4 cases) during 2003–2010, after implementation of the 2003 regulation (p = 0.0005). The median annual number of deaths dropped from 2.5 (range 1–6; total 38 deaths) to 0 (range 0–1; total 1 death) after implementation of the 2003 regulation (p = 0.0001).

**Figure 1:**
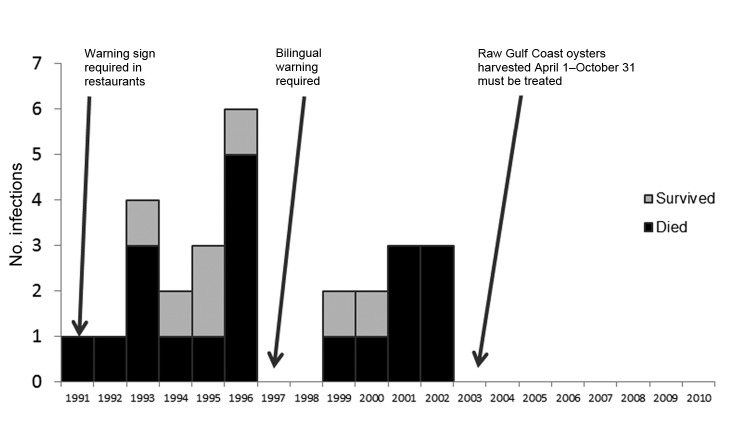
*Vibrio vulnificus* infections among 27 California patients who consumed only raw oysters, by year, 1991–2010. Arrows indicate enactment of different requirements.

Twenty-seven case-patients with foodborne *V. vulnificus* infection reported consuming only raw oysters (i.e., no other seafood); 20 (74%) of these patients died. The median annual number of patients who consumed only raw oysters dropped from 2 (range 0 to 6) during 1991–2002, before implementation, to 0 (none in the entire time) during 2003–2010, after implementation of the 2003 regulation (p = 0.0005) (Figure 1). The median annual number of deaths among patients who consumed only raw oysters decreased from 1 (range 0 to 5) to 0 (none in the entire time) after implementation of the 2003 regulation (p = 0.0005).

The 27 patients who consumed only raw oysters had a median age of 48 years (range 27–72); 24 (89%) were men, and 23 (85%) were Hispanic. All had an underlying condition predisposing them to severe infection, including 22 (81%) with liver disease, cirrhosis, or hepatitis. The oyster harvest site was known (for 19) or suspected (for 2) for 21 (78%) patients who consumed only raw oysters; all oysters were traced to the Gulf of Mexico.

**Figure 2:**
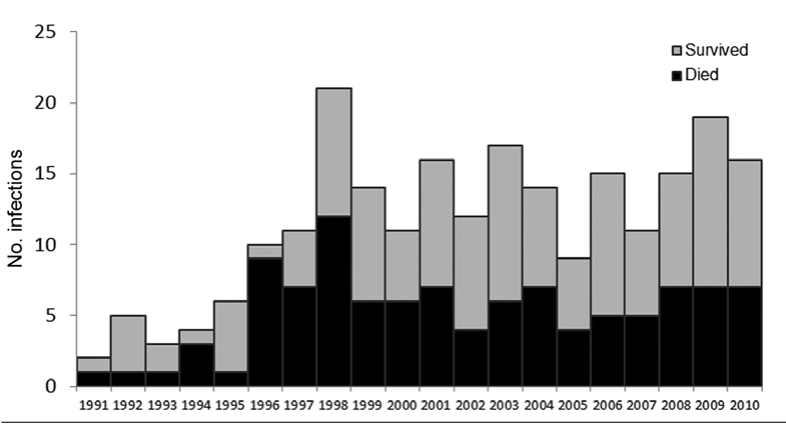
*Vibrio vulnificus* infections among 231 persons who consumed only raw oysters, by year, United States (excluding California), 1991–2010.

During 1991–2010, states other than California reported 231 cases of foodborne *V. vulnificus* infection in patients who reported consuming only raw oysters; 106 (46%) of these patients died. The median annual number of non-California patients who reported consuming only raw oysters was 10.5 (range 2–21) during 1991–2002 and 15 (range 9–19) during 2003–2010 (p = 0.02) (Figure 2). The median annual number of these non-California patients who died was 5 (range 1–12) during 1991–2002 and 6.5 (range 4–7) during 2003–2010 (p = 0.17). The oyster harvest site was known for 151 (65%) of these patients; 145 (96%) of the oysters were traced to the Gulf of Mexico.

## Discussion

The data strongly suggest that the dramatic and sustained drop in reported raw oyster–associated *V. vulnificus* illnesses and deaths in California was related to the 2003 California regulation that restricts the sale of raw oysters harvested from the Gulf Coast during the 7 warmest months to oysters treated with postharvest processing. This conclusion is supported by the lack of decline after 2002 in the number of foodborne *V. vulnificus* cases and deaths associated with consuming only raw oysters among persons living in other states, none of which has a similar raw oyster restriction. The significant reduction after 2002 in the larger number of California patients who consumed raw or cooked oysters, with or without other seafood, suggests that many of these illnesses were also due to raw oysters.

Evidence suggests that the proportion of persons eating raw oysters in California did not decrease after the 2003 regulation. Surveys of persons in the California counties of Alameda, Contra Costa, and San Francisco who participated in the Foodborne Diseases Active Surveillance Network (FoodNet) showed that in 2006–2007, ≈2% of persons interviewed reported eating raw oysters in the previous 7 days (*13*), compared with 2% in 2002–2003 (*14*). The FoodNet surveys also did not show any significant difference between the proportion of Hispanic and non-Hispanic White persons who reported eating raw oysters. Thus, it is not known why the proportion of case-patients who were Hispanic (85%) was much higher than the proportion of the state’s Hispanic population (32% in 2000 US Census [*15*]). The higher prevalence of chronic liver disease among the Hispanic populations may be a contributing factor (*16*).

To decrease the risk of *V. vulnificus* infection, persons in high-risk groups and others who want to decrease the risk of illness should not eat raw, unprocessed oysters, especially those harvested from the Gulf Coast during the summer months. Summer-harvested oysters from the Mid-Atlantic region, however, should also be of concern because they have been shown to have *V. vulnificus* levels nearly as high as those from the Gulf Coast (*7**,**8*). Persons at high risk for disease should also avoid seawater exposure if they have a fresh wound and should seek medical care as soon as possible if signs of wound infection develop after such exposure. Clinicians’ high awareness of the risk factors for *V. vulnificus* infection along with prompt diagnosis and treatment can substantially improve patient outcomes (*2**–**5*).

Our study had some limitations. First, the surveillance system is based on passive reporting, and some cases might not have been reported. If cases occurred after 2003 that were not reported to public health, the decline might not have been so significant. However, any underreporting would most likely have occurred both before and after 2003, and *V. vulnificus* disease is severe enough that most cases are likely recognized and reported. Second, because vibriosis did not become officially nationally reportable until 2007, some of the increase of reported cases nationally after 2002 could have been due to increased reporting. All states, however, have been voluntarily reporting vibriosis since before 2003, and FoodNet population-based surveillance data, albeit based on a smaller national catchment area, also showed increased incidence of *V. vulnificus* cases during 1996–2010 (*1*). Furthermore, although we show a significant drop in *V. vulnificus* cases for which patients had only raw oyster exposure in California after implementation of the 2003 regulation, a small but undefined risk for *V. vulnificus* infection remains among persons in California who eat raw oysters.

A variety of approaches have been used to address oyster-associated cases of severe *V. vulnificus* infection and those that lead to death, including consumer education, time and temperature control regulations for raw oysters, and postharvest processing. In 2001, the Interstate Shellfish Sanitation Conference (a national organization with participants from the US Food and Drug Administration, the US Environmental Protection Agency, the shellfish industry, Gulf Coast states, and others), as part of its proposed *Vibrio vulnificus* Risk Management Plan, pushed to increase education of at-risk oyster consumers in participating states (*17*). In 2004, an Interstate Shellfish Sanitation Conference survey of raw oyster consumers in California, Florida, Louisiana, and Texas “found no significant increase in overall consumer knowledge about the risk of eating raw oysters or the proportion of high-risk consumers who stopped eating them” when compared with results of a similar survey in 2002 (*18*). In May 2010, time- and temperature-control regulations (e.g., within how many hours after harvest oysters must be refrigerated and cooled) were enacted in Florida, Louisiana, and Texas, but compliance has not been evaluated (*18*).

Educational outreach to high-risk populations is a time-honored public health approach, and some have credited that approach with success in reducing the incidence of vibriosis associated with raw oyster consumption, such as in Florida (*19*). However, the survey of raw oyster consumers mentioned above suggests difficulty in reaching or convincing high-risk consumers. Implementation of California’s warning regulations was not followed by a reduction in the number of reported cases or deaths caused by *V. vulnificus*. The higher than expected proportion of Hispanic patients also suggests that the 1997 regulation to reach Spanish-speaking consumers was not effective. Not until after the 2003 emergency regulation was implemented did the number of cases and deaths drop significantly. A similar regulation to restrict the sale of raw summer-harvested Gulf Coast oysters to those treated by postharvest processing, if implemented nationwide, would likely decrease *V. vulnificus* illnesses and deaths due to eating unprocessed raw oysters.
